# An epithelial-mesenchymal transition-related long non-coding RNA signature to predict overall survival and immune microenvironment in kidney renal clear cell carcinoma

**DOI:** 10.1080/21655979.2021.1880718

**Published:** 2021-02-10

**Authors:** You-Peng Zhang, Yong-Biao Cheng, Sen Li, Ning Zhao, Zhao-Hui Zhu

**Affiliations:** a Department of Urology, Union Hospital, Tongji Medical College, Huazhong University of Science and Technology, Wuhan, China; b Department of Cardiology, Union Hospital, Tongji Medical College, Huazhong University of Science and Technology, Wuhan, China

**Keywords:** Kidney renal clear cell carcinoma, epithelial-mesenchymal transition, lncRNAs, data mining

## Abstract

Kidney renal clear cell carcinoma (ccRCC) is a malignant tumor originating from renal tubular epithelium, lncRNAs can regulate the occurrence and development of EMT by targeting EMT transcription factors. We constructed a new survival signature based on EMT-related differentially expressed lncRNAs obtained from the Cancer Genome Atlas (TCGA-KIRC). We first determined 1377 EMT-related lncRNAs, lncRNA AL035661.1 with the largest correlation coefficient and the target gene was *PFN2* (cor = 0.843; *P*= 1.37E-146). Meanwhile, we found an AUC of 0.758 in our signature and we predicted the AUC values of the patients’ 1, 2, 3-year survival rate as 0.768, 0.749, and 0.762 in TCGA cohort, respectively. Multivariate COX analysis was performed to determine if risk score was an independent prognostic predictor of OS. The results indicated that our risk score can be an independent predictor for OS (Univariate: HR = 1.350, 95% CI = 1.276–1.428, *P*< 0.001; Multivariate: HR = 1.295, 95% CI = 1.201–1.396, *P*< 0.001). We identified novel EMT-related lncRNAs markers for ccRCC prognosis. The underlying mechanism between EMT-related lncRNAs in ccRCC and tumor immunity is still unclear and requires further study.

## Background

Kidney renal clear cell carcinoma (KIRC) is a malignant tumor originating from renal tubular epithelium, accounting for approximately 85% of kidney cancer and claiming over 14,000 lives per year in the United States [1,2]. Coupled with relapsing metastases in a quarter of patients with ‘local’ disease after curative nephrectomy, highest metastasis and mortality constitutes a substantial medical burden [3]. With the advancement of tumor detection methods and treatment methods, the survival rate of patients with KIRC has been significantly improved, but the overall survival (OS) and progression-free survival are still low. More than 1/3 of the diseases have metastasized at diagnosis [4]. TNM stage is still the most critical prognostic factor for KIRC. However, due to tumor heterogeneity, even in the same stage, the survival rate of patients will be very different. There is a pressing need for new early diagnosis markers and treatment points to improve the survival and prognosis of patients.

Epithelial-mesenchymal transition (EMT) has received widespread attention for its potential role in transforming benign tumors into aggressive and metastatic tumors [5]. It is a complex and reversible process in which cell phenotypes, functions and a large number of molecules are expressed Changes. EMT is a physiological process necessary for normal embryonic development. EMT is a dynamic process, and multiple cell signal transduction pathways are involved in regulating its occurrence and changes. At present, researchers believed that TGF-β is the most relevant cytokine in EMT, and many transcription factors such as ZEB1/2, bHLH protein (Twist), and Snail family (Snail, Slug) are also involved [6]. Under hypoxic conditions, tumor cells directly or indirectly (by up-regulating the expression of TGF-β) promote the expression of EMT transcription repressor Snail through hypoxia-inducible factors [7]. By activating the epidermal growth factor receptor (EGFR) signaling pathway, it induces tumor cells to develop EMT.

Long non-coding RNA (lncRNA) belonged to RNA molecule with a transcript length of over 200 nt and regulate the expression level of genes. lncRNA participates in various biological regulatory processes, and is closely linked to the occurrence, development and metastasis of tumors. lncRNA is a transcript that lacks the potential to encode a protein, but shows key locations such as antigen exposure, recognition, and immune infiltration. Therefore, the potential of lncRNA in predicting tumor progression and OS has attracted more and more attention. A recent study reported that lncRNA LINC01234 knockdown impaired ccRCC cell proliferation, migration and invasion in vitro and inhibited the EMT process [8]. Similarly, LncRNA FGD5-AS1 can competitively interact with mir-5590-3p to control the downstream signaling pathway of ERK/AKT and enhance the malignancy of tumors [9]. With the advanced of microarray and sequencing technologies, multiple genome databases have been established. In addition, some studies used the corresponding genome database to determine EMT-associated lncRNAs (EALs) with prognostic value among tumor tissue and normal tissue, so as to establish prognostic signatures for a variety of cancers. However, the prognostic value of ccRCC has not been fully studied to a large extent.

Thus, we hypothesis that EALs plays a critical role in KIRC and we aimed to construct a new survival signature based on EMT-related differentially expressed lncRNAs (DELs) got from the Cancer Genome Atlas (TCGA-KIRC) to display dysfunctional lncRNAs microenvironments and provide useful lncRNAs biomarkers.

## Methods

### Data acquisition and analysis

The RNA sequencing data and clinical information of 611 KIRC (72 normal and 539 tumor) patients were got from the TCGA. Use the scale method provided by the ‘limma’ R package to normalize the gene expression profile. The collected clinical data included gender, age, stage, TMN classification. In total 200 EMT-associated genes (EAGs) were download through the ‘HALLMARK_EPITHELIAL_MESENCHYMAL_TRANSITION’ gene set based on the Gene set enrichment analysis (GSEA). Pearson correlation was used to calculate the correlation among the EMT-related genes and lncRNAs. An EMT-related lncRNA considered with a correlation coefficient |*R^2^*|>0.3 and *P*< 0.001.

### Construction of EMT-associated lncRNAs signature

Univariate Cox analysis was used to identify EALs accompanied by prognostic data. The Cytoscape generates an interactive network of EMT-associated and lncRNAs. Lasso-Penalized Cox regression analysis was used to build a prognostic signature. The penalty parameters (λ) of the model were decided by cross-validation following a minimum standard of tenfold. The patient risk score was also calculated. The risk score was acquired based on the formula: risk score = e^sum (lncRNA’s expression×coefficient)^. Then, the patients were randomly divided into the high-/low-risk group based on the risk score median of the risk score value. PCA and t-SNE were varied out to analysis the distribution of distinct subgroups. Kaplan–Meier analysis to explore the OS and ROC analysis was applied to estimate the prognostic signature.

### Enrichment functional analysis

Kyoto Encyclopedia of Genes and Genomes (KEGG) and Gene Ontology (GO) analyses applying the ‘clusterProfiler’ R package to EMT-related differentially expressed (DEGs). Meanwhile, single-sample gene set enrichment analysis (ssGSEA) used to evaluate the activity of 13 immune-related pathways and the infiltrating score of 16 immune cells based on the ‘GSVA’ R package. Gene set enrichment analysis (GSEA) was applied to elucidate gene expression data. GSEA can determine whether a predefined set of genes can show significant differences in consistency between two biological states

### Statistical analysis

Statistical analyses were applied using R version 3.5.3. The unpaired Student’s t-test and the Wilcoxon test were used to evaluate the normal distribution variables and the non-normal distribution variables, respectively. The Benjamin–Hochberg method converts the *P* value to FDR. For each statistical analysis, a *p *≤ 0.05 was statistically significant.

## Results

### Functional analyses

**Figure 1.**
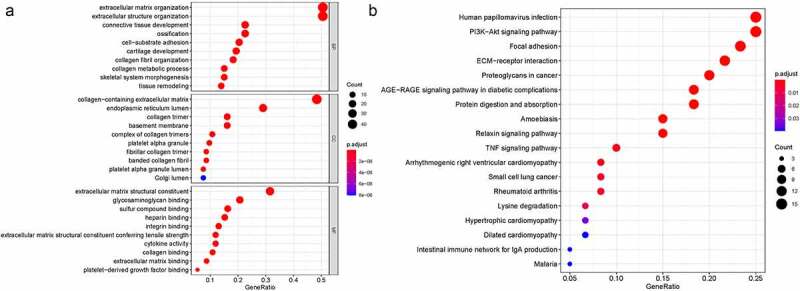
The biological functions and signaling pathways of the 93 genes in TCGA. A. GO; B. KEGG

We first identified 93 (71 upregulated and 22 downregulated) DEGs in TCGA-KIRC and analysis these 93 genes biological functions and signaling pathways. Biological process (BP) were mainly enriched in extracellular matrix organization, extracellular structure organization, collagen fibril organization, connective tissue development. Cellular component (CC) were mainly enriched in collagen-containing extracellular matrix, endoplasmic reticulum lumen, collagen trimer. Molecular function (MF) were mainly enriched in extracellular matrix structural constituent, glycosaminoglycan binding, heparin binding, collagen binding. KEGG were mainly enriched in ECM-receptor interaction, focal adhesion, proteoglycans in cancer, PI3K-Akt signaling pathway, relaxin signaling pathway, TNF signaling pathway Figure 1 and **Table S1, S2**. The DEGs were clearly enriched in EMT-association signaling pathways and biological course.

### Prognostic EMT-related lncRNAs in ccRCC

According to the screening criteria, we determined 1377 EMT-related lncRNAs based on the 200 EAGs, lncRNA AL035661.1 with the largest correlation coefficient and the target gene was *PFN2* (cor = 0.843; *P*= 1.37E-146; **Table S3**). To screen the prognostic lncRNAs, differently expressed lncRNAs were conducted in univariate COX analysis. Then, 562 lncRNAs of great significance in univariate COX analysis were comprised in multivariate COX analysis **Table S4**. As a result, 18 differently expressed lncRNAs (DLGAP1.AS2, AC095057.3, AC103706.1, MIR193BHG, AC026401.3, HOXB.AS4, DBH.AS1, AC005261.3, CD27.AS1, AL365203.2, LINC00460, FOXD2.AS1, AC084876.1, AC121338.2, EMX2OS, AC002070.1, LINC01550, and EPB41L4A.DT) were selected as independent prognosis factors of ccRCC patients **Table S5**. In addition, in our study cohort, the risk score of individual patients was calculated. The cohorts were divided into two (high – and low-risk) groups reference the median risk score value.

### Building the prognostic DELs signature

**Figure 2.**
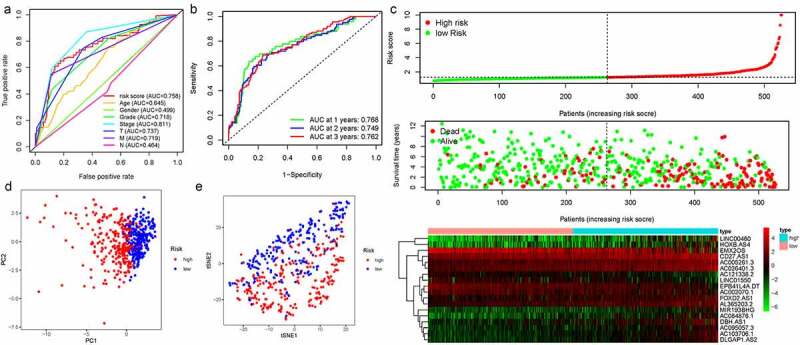
The results of EMT-related gene signature based on TCGA. A. the AUC values of the risk factors; B. the AUC values of the patients’ 1, 3, 5-year survival rate; C. risk score distribution map and the survival status map; D. PCA analysis; E. t-SNE analysis

**Figure 3.**
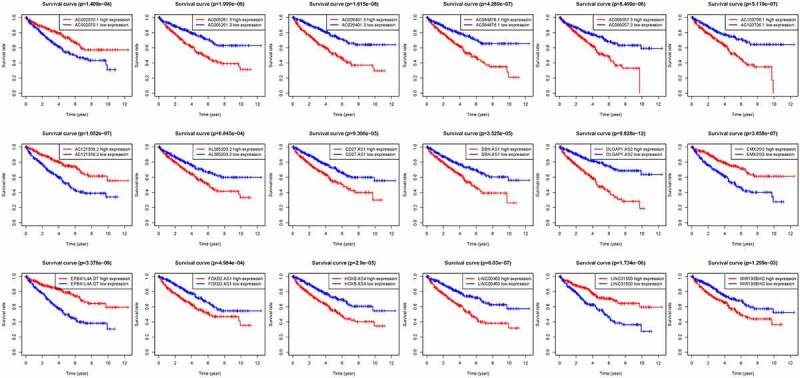
Kaplan-Meier results demonstrated that 18 DELs expression significance effect patient’s OS

**Figure 4.**
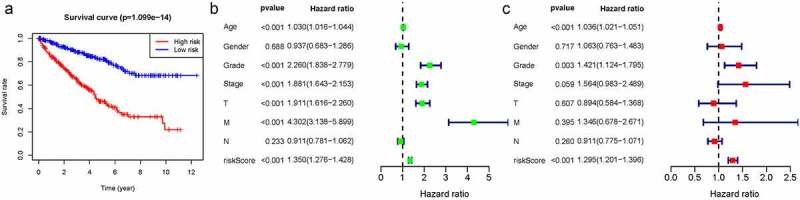
Kaplan-Meier results of our risk model and univariate/multivariate cox analysis to determine if risk score was an independent prognostic predictor of OS. A. Kaplan-Meier results; B. univariate; C. multivariate

We thus construct the risk score based on the above formula. Meanwhile, we found an area under curve (AUC) of 0.758 in our signature and we predicted the AUC values of the patients’ 1, 2, 3-year survival rate as 0.768, 0.749, and 0.762 in TCGA cohort, respectively, Figure 2a,2b. Risk score distribution map and the survival status map were shown in Figure 2c, the results indicated that high risk patients had a higher possibility of decease when compared those with low risk in TCGA cohort. PCA and t-SNE results suggested the patients in distinct risk groups were separated into two directions in TCGA cohort Figure 2d,2e. Kaplan–Meier results demonstrated that 18 DELs expression significance effect patient’s OS Figure 3. Meanwhile, the high-risk group displayed a poorer survival outcome when compared to the low-risk group in TCGA cohort (*p*= 1.099e−14) Figure 4a. Available variables were performed to determine if risk score was an independent prognostic predictor of OS. The results indicated that our risk score can be an independent predictor for OS (Univariate: HR = 1.350, 95% CI = 1.276–1.428, *P*< 0.001; Multivariate: HR = 1.295, 95% CI = 1.201–1.396, *P*< 0.001 Figure 4b,4c).

### Construction of the predictive nomogram

**Figure 5.**
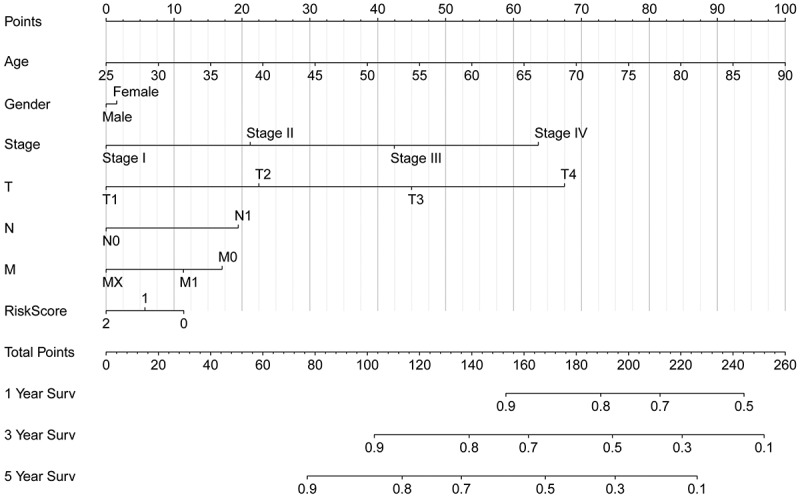
The nomogram was constructed from the clinicopathological data as well as the developed prognostic model

The nomogram was constructed from the clinicopathological data as well as the developed prognostic model. The nomogram involved seven items in TCGA: age, sex, grade, stage, T, M, and N Figure 5. Based on our prognostic signature with clinical factors analysis fortified the prediction specificity and sensitivity for 1‐, 3‐, and 5‐year OS, thus increasing the usefulness in the clinical management of patients.

### Gene set enrichment analysis

**Figure 6.**
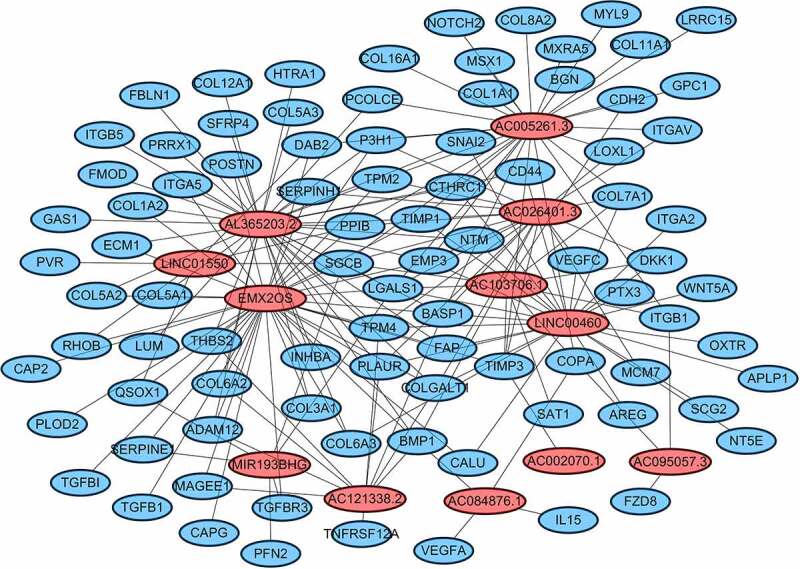
Cytoscape explored the potential link between lncRNA and target genes

**Figure 7.**
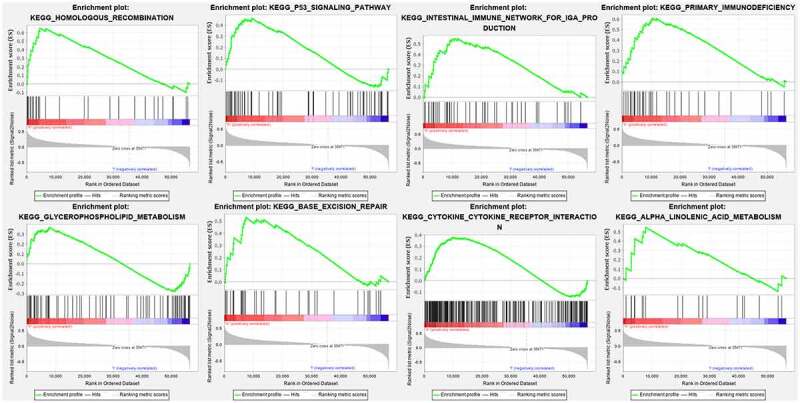
Functional annotation was performed through GSEA among high risk group

Cytoscape explored the potential link between lncRNA and target genes Figure 6. Functional annotation was performed through GSEA between high- and low-risk group. The results suggested that the high-risk groups were mainly enriched in the tumor-related pathways involving in HOMOLOGOUS_RECOMBINATION, P53_SIGNALING_PATHWAY, INTESTINAL_IMMUNE_NETWORK_FOR_IGA_PRODUCTION, PRIMARY_IMMUNODEFICIENCY, GLYCEROPHOSPHOLIPID_METABOLISM, BASE_EXCISION_REPAIR, CYTOKINE_CYTOKINE_RECEPTOR_INTERACTION, ALPHA_LINOLENIC_ACID_METABOLISM Figure 7.

### Immune microenvironment analysis in TCGA cohort

**Figure 8.**
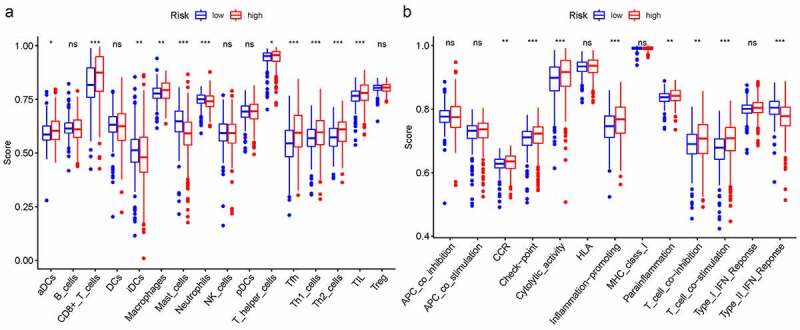
The relationship between immune status and risk scores with ssGSEA. A. immune function; B. immune cells

In order to further explore the connection between immune status and risk scores, we quantified enrichment scores for special immune cell subsets and connected biological functions or signaling pathways of ssGSEA. The results suggested that the score of aDCs, CD8 + T cells, iDCs, macrophages, mast cells, neutrophils, T helper cells, Tfh, Th1/Th2 cells, and TIL were also significantly different Figure 8a. Moreover, the score of CCR, check point, cytolytic activity, inflammation promoting, parainflammation, T cell co-inhibition/co – stimulation, and type II IFN response were significantly different among the high- and low-risk group Figure 8b.

## Discussion

Changes in gene expression during EMT lead to many phenotypic changes, such as changes in cell morphology, loss of adhesion, and acquisition of stem cell-like features. LncRNA can regulate the process of EMT, so exploring the potential molecular mechanism of EMT in kidney cancer may be of great significance for inhibiting the progression of kidney cancer and improving the clinical prognosis of patients with kidney cancer. In this study, we comprehensive determined the expression of EMT-related differentially expressed lncRNAs in kidney renal clear cell carcinoma tissues and their relationship with OS. A new prognostic signature incorporating 18 DELs has been constructed. Functional analysis showed enrichment of tumor-related pathways. Therefore, this study informs on the potential biomarkers and therapeutic targets of the DELs signaling pathways.

In this study, we first identified 18 DELs (DLGAP1.AS2, AC095057.3, AC103706.1, MIR193BHG, AC026401.3, HOXB.AS4, DBH.AS1, AC005261.3, CD27.AS1, AL365203.2, LINC00460, FOXD2.AS1, AC084876.1, AC121338.2, EMX2OS, AC002070.1, LINC01550, and EPB41L4A.DT) as independent prognostic factors for KIRC. A recent study reported that DLGAP1-AS2 knockdown may suppress hepatocellular carcinoma cell migration and invasion through regulating miR-154-5p methylation [10]. Similarly, DLGAP1-AS2 modulated glioma cell proliferation, migration and apoptosis by regulating YAP1 [11]. LincNORS is a splicered lincRNA generated at MIR193BHG site, this lincRNA fine-tunes cytosterol/steroid biosynthetic by inhibiting the expression of multiple pathway components [12]. Genome-wide screening found HOXB-AS4 as specifically methylated in pancreatic cancer cells [13].

Study indicated that DBH-AS1 can increase glycolytic activity in melanoma cells, thus disrupting melanoma progression through miR-223-3p/EGFR/AKT axis [14]. Meanwhile, DBH-AS1 may serve as an oncogenic lncRNA via modulating the PI3K/Akt pathway in osteosarcoma [15]. Study identified autophagy-related lncRNAs CD27.AS1 to constructed a prognostic signature in colon adenocarcinoma [16]. LINC00460 was usually upregulated in cervical cancer tissues and cell lines, knockdown of LINC00460 restrain cervical cancer cell growth and invasion in vitro and attenuated tumorigenesis in vivo [17]. Overexpressed FOXD2-AS1 is linked to tumor size and TNM stage and a risk factor of OS and disease-free survival (DFS) in cancer patients [18]. EMX2OS is a key metabolism-related enhancer RNA in KIRC with a favorable impact on survival [19] and EMX2OS is regulated by TCF12 transcription and co-regulates the proliferation, migration and invasion of prostate cancer cells with FUS protein by activating cGMP-PKG pathway [20]. EPB41L4A-AS2 can suppressed tumor cell proliferation in breast, renal, and lung cancer cell lines [21].

EMT is induced by a network of transcription factors (EMT-TFs). Snail, TWIST, and ZEB are considered to be the core transcription factors of EMT, which can directly or indirectly inhibit the expression of epithelial markers to initiate EMT [22]. Recent studies have reported that a variety of lncRNAs regulate the occurrence and development of EMT by targeting EMT transcription factors, but the potential regulatory mechanism between tumor immunity and EMT is still unclear. We also found that the high-risk group were mainly enriched in immune and cancer-related biological processes and signaling pathways such as immune network for IgA production, primary immunodeficiency. So, we further explore the relationship between immune status and risk scores. The advent of checkpoint inhibitors has revolutionized the systemic treatment of many malignancies, including renal cell carcinoma (RCC), and in clinical trials, a variety of PD-1, PD-L1, and CTLA-4 inhibitors have been shown to respond to patients and improve patient survival [23]. The imbalance of Th1/Th2 and Th17/Treg cells was linked to the tumor size, lymph node metastases, and vasoinvasion [24]. In general, weakened anti-tumor immunity in high-risk patients may be one of the reasons for their poor prognosis.

In this study, a reliable prognostic risk model for ccRCC patients was established using DELs sequencing data and clinical information from the TCGA database. Therefore, compared with other clinicopathological features, the prognostic function of our risk model is very satisfactory. But, our work has to prove in further independent cohorts and predictive DELs functional by experiments.

In addition, our results do not provide an accurate clinical data as the relatively small sample size of patients.

## Conclusion

We identified novel epithelial-mesenchymal transition-related lncRNAs biomarkers markers for ccRCC prognosis. The underlying mechanism between epithelial-mesenchymal transition-related lncRNAs in ccRCC and tumor immunity is still unclear and requires further study.

## Supplementary Material

Supplemental Material

## Data Availability

Data sharing is not applicable to this article as no datasets were generated or analyzed during the current study.
